# Accounting for NAD Concentrations in Genome-Scale Metabolic Models Captures Important Metabolic Alterations in NAD-Depleted Systems

**DOI:** 10.3390/biom14050602

**Published:** 2024-05-20

**Authors:** Roland Sauter, Suraj Sharma, Ines Heiland

**Affiliations:** 1 Department of Arctic and Marine Biology, UiT The Arctic University of Norway, 9019 Tromsø, Norway; roland.sauter@uit.no; 2 Department of Biomedicine, University of Bergen, 5020 Bergen, Norway; suraj.sharma@uib.no; 3 Department of Clinical Medicine, University of Bergen, 5020 Bergen, Norway

**Keywords:** NAD, proteomics, constraint-based model, genome-scale metabolic modeling, proteomics integration, FBA

## Abstract

Nicotinamide adenine dinucleotide (NAD) is a ubiquitous molecule found within all cells, acting as a crucial coenzyme in numerous metabolic reactions. It plays a vital role in energy metabolism, cellular signaling, and DNA repair. Notably, NAD levels decline naturally with age, and this decline is associated with the development of various age-related diseases. Despite this established link, current genome-scale metabolic models, which offer powerful tools for understanding cellular metabolism, do not account for the dynamic changes in NAD concentration. This impedes our understanding of a fluctuating NAD level’s impact on cellular metabolism and its contribution to age-related pathologies. To bridge this gap in our knowledge, we have devised a novel method that integrates altered NAD concentration into genome-scale models of human metabolism. This approach allows us to accurately reflect the changes in fatty acid metabolism, glycolysis, and oxidative phosphorylation observed experimentally in an engineered human cell line with a compromised level of subcellular NAD.

## 1. Introduction

Nicotinamide adenine dinucleotide (NAD) and its phosphorylated form NADP are essential metabolic cofactors. As such, they serve as electron acceptors and donors and are reversibly interconverted between the oxidized NAD(P)^+^ and the reduced forms NAD(P)H. These redox reactions do not change the amount of the NAD(P) moiety. However, there are numerous signaling reactions that consume NAD and release its precursor nicotinamide (Nam). These include, but are not limited to, protein deacetylation by sirtuins and mono- and poly-ADP-ribosylation of proteins [1,2,3,4,5]. These NAD-dependent signaling reactions are involved in a wide range of cellular processes [5,6,7,8,9] such as epigenetic regulation [10,11], DNA damage recognition [12,13], regulation of enzyme activity [11], and calcium signaling [14,15,16]. The NAD consumption flux caused by NAD signaling reactions is surprisingly high, resulting in cell-type-specific half-lives from 15 min to a few hours [17,18]. The cellular NAD levels, therefore, need to be constantly replenished. If the balance between NAD synthesis and consumption is disturbed, NAD levels decline [19,20] and affect a wide range of metabolic and signaling reactions. This is often observed during aging [10,20,21].

Metabolic modeling approaches are a valuable tool for the investigation of metabolic alterations. Constraint-based modeling (CBM) is a popular approach for simulating genome-scale metabolic changes. CBM formulates metabolism as a convex optimization problem, where metabolic fluxes are set to optimize an objective function (often biomass or ATP production) under constraints such as mass conservation, steady state, and measured uptake rates, as well as maximum and minimum reaction fluxes (flux boundaries) [22,23]. Flux boundaries are often derived from expression data [22,23,24,25,26]. Flux balance analysis (FBA) is the most common variant of CBM and has been used for a wide range of applications [27] from basic analyses of single pathways [28] to complex genome-scale models of the human metabolism [29]. However, FBA solutions can have multiple possible flux distributions that achieve the same objective. A plethora of FBA variants and tools that enable FBA exist [30,31,32,33,34], of which two commonly used variants are flux variability analysis [35] (FVA) and parsimonious FBA [36] (pFBA). Both built on a previously solved FBA problem to make further predictions. In FVA, the solution space is described for a given fraction of the optimal objective value [35]. This helps identify essential reactions, reactions with a limited flux variability, and reactions with a high flux variability, indicating the cell has alternative routes to achieving the same outcome. On the other hand, pFBA aims to find the most parsimonious (in terms of total flux) solution among the possible FBA solutions. A second optimization is run, where the objective flux is constrained and the sum of the magnitudes of all fluxes is minimized [36]. By minimizing flux, next to maximizing the flux through the objective function, pFBA solutions represent metabolic states that require the least cellular investment to maintain the desired objective.

In current state-of-the-art genome-scale metabolic models (GSMMs), cofactor concentrations are generally not explicitly considered. This is because most GSMMs employ an FBA approach that focuses on the stoichiometry and thermodynamic feasibility of reactions, rather than the absolute concentration of metabolites or cofactors. Cofactors like NAD are usually represented solely based on the interconversion between their oxidized and reduced states in these models, with the steady-state constraint balancing interconversions between cofactor states. GSMMs usually do not account for concentration changes of cofactors due to the breakdown or biosynthesis of the cofactor moiety. Implementing the kinetic effects of concentration changes in cofactor moieties is more challenging than accounting for changes in carbon sources as these changes impact a larger set of enzymes. Based on the KEGG database [37], 16% of all metabolic reactions in humans are dependent on either NAD(H) or NADP(H). Among human metabolic models, more than 10% of reactions in Recon3D [29] and 20% of reactions in the MitoCore model [38] involve NAD(H) or NADP(H).

Changes in the cofactor moiety have been shown to have major implications for disease progression, and this has been successfully replicated by a modeling approach that aimed to simulate the effects of flavin adenine dinucleotide (FAD) biosynthesis diseases [39]. The authors demonstrated that correct FAD representation and accounting for its biosynthesis and degradation is crucial for modeling diseases relating to multiple-acyl-CoA-dehydrogenase deficiency and the systemic depletion of FAD. As NAD is a more common cofactor, it is even more important to be able to correctly simulate effects of changes in the concentration of the NAD moiety. In contrast to FAD, which is commonly covalently bound to enzymes, NAD binding to enzymes is usually reversible and dependent on its concentration and the binding affinity. Therefore, the approach used to simulate changes in FAD concentrations cannot be extended to include NAD, but a different approach is needed to investigate the effect of NAD concentrations. A critical parameter is the affinity of NAD-dependent enzymes for NAD in relation to the free NAD concentration. The Michaelis–Menten constant ($K_{m}$) describes the concentration at which half of the enzyme’s active sites are occupied. Enzymes with low $K_{m}$ values have high affinities, while enzymes with high $K_{m}$ values have low affinities.

In this paper, we present a genome-scale metabolic modeling approach that parameterizes metabolic models by using proteomics data and incorporates the concentrations of free NAD to further investigate the effects of NAD concentrations. To this end, we used a proteome dataset from an engineered human cell line with a compromised subcellular NAD availability. The predictions were compared to the corresponding experiments.

## 2. Materials and Methods

### 2.1. Metabolic Model

We used the MitoCore [38] model of human core carbon metabolism for all the analyses presented in this paper. MitoCore encompasses 324 metabolic reactions, 83 transport steps between mitochondrion and cytosol, and 74 metabolite inputs and outputs. It offers several key advantages. Firstly, it effectively divides metabolism between the cytosolic and mitochondrial matrix, enabling a more precise representation of cellular processes. Secondly, it enhances the modeling of connecting transport steps, ensuring accurate accounting of metabolite exchange between compartments. Thirdly, MitoCore distinguishes between prosthetic groups and free cofactors in reactions, crucial for understanding metabolic pathways. Lastly, the model introduces a novel representation of the respiratory chain and the proton motive force, enhancing our understanding of energy metabolism within mitochondria. As the objective function, we use MitoCore’s included ATP demand objective function [38].

### 2.2. Proteomics Data

A cell line expressing PARP1 targeted to mitochondria has been used [40,41,42,43]. Proteins and peptides were quantified by VanLinden et al. [41] via an MS/MS-based approach using tandem mass tags [44]. All analyses of cell lines were performed in triplicates, with abundance ratios calculated for each replicate by comparing its measurement to a reference sample, resulting in a dataset containing 6392 proteins.

### 2.3. Construction of Cell-Line-Specific Models

To construct models that represent parental HEK293 and 293mitoPARP cells, we used the proteome data from the respective cell lines. First, we calculated the mean abundance ratio for each protein across the 3 replicates of each cell line. These resulting values, corresponding to 269 out of 6392 proteins, were then mapped onto model reactions using the COBRA toolbox [45], and reaction scores were obtained. Subsequently, we created cell-line-specific models by integrating these reaction scores into the MitoCore model using GIMME [46]. For GIMME’s activity threshold, we calculated the median reaction scores for both the cell lines and used the median of those values. The value for GIMME’s minimum required objective fraction was set to 0.8, as the resulting models were stable for a wide range of values around this threshold (cf. Figure S3). Finally, we obtained two models representing parental HEK293 and 293mitoPARP cells, containing 470 and 462 reactions, respectively. Reaction mapping and integration with GIMME were performed using the COBRA toolbox 3.0 [45] in Matlab2019a [47]. Gurobi 9.0.2 (Gurobi Optimization, LLC, Beaverton, OR, USA) [48] was used as a solver for linear optimization.

### 2.4. Model Parameterization for Different NAD Concentrations

We further parameterized both HEK293 and 293mitoPARP models for both parental and decreased NAD (as well as NADH, NADP, and NADPH) concentrations. This involved extracting the Michaelis–Menten constant ($K_{m}$) values of enzymes for NAD(H) and NADP(H) from the Brenda enzyme database [49]. We successfully mapped $K_{m}$ values to 90 out of 104 reactions that involve NAD(H) and NADP(H) in the MitoCore model. In case where human $K_{m}$ values were not available, we adopted values from other mammals in the following order: pig (*Sus scrofa domesticus*), cow (*Bos taurus*), rat (*Rattus norvegicus*), and mouse (*Mus musculus*). When multiple $K_{m}$ values from the same species were available, we used the minimum $K_{m}$ value. This approach resulted in more conservative estimates of the effect, given that a lower $K_{m}$ value leads to a less constrained flux boundary (cf. Figure S4). The parameterization of NAD-dependent models was conducted using Python 3.10.11 [50] with COBRApy 0.26.3 [51] and Gurobi (Gurobi Optimization, LLC, Beaverton, OR, USA) 9.0.2 [48].

We used the cell-line-specific models and adjusted the flux boundaries according to the acquired $K_{m}$ values. First, we performed a flux variability analysis (FVA) to determine the maximum and minimum flux for reactions (see Figure S5 for the selection of fractional optimality). Based on the obtained feasible flux ranges ($\underline{v\;}\leq v\leq \bar{v\;}$), we derived the new upper bounds for a cofactor concentration C from the upper flux ranges as follows:

(1) $$\bar{v_{C}}=\bar{v\;}\cdot \frac{C}{K_{m}+C}\cdot s\;$$

The scaling factor s with wild-type NAD concentration ($C_{0}$) is defined as:

(2) $$s=\frac{K_{m}+C_{0}}{C_{0}}$$

The scaling factor allows us to correct for the case where the models do not assume full cofactor saturation of all enzymes by normalizing to the reaction flux under condition $C_{0}$, which is 0.11 mM in the cytosol and 0.23 mM in the mitochondria (see Table 1) [40,41,52,53]. NADP(H) was treated identically to NAD(H); i.e., the same concentrations were used for NADP(H)-dependent reactions. For most of the enzymes mapped to the model, the parental $C_{0}$ is close to the mapped $K_{m}$ and far from saturation (see Figure S6). Similarly, we calculate the new value of the lower limit of the flux using $\underline{v\;}$ as:

(3) $$\underline{v_{c}}=\underline{v}\cdot \frac{C}{K_{m}+C}\cdot s$$

If the FVA range is zero for both lower and upper bound, $K_{m}$-based adjustment is without effect and the respective reaction remains blocked by the model constraints. All other cofactor-dependent reactions can potentially carry NAD-concentration-constrained flux in an FBA solution according to Equations (1) and (3).

### 2.5. Pathway Analysis

Once the models were parameterized for their respective free NAD concentrations, we analyzed different metabolic pathways. To derive minimal flux values, we performed pFBA with the fraction of optimum set at 100%. The pFBA solutions enabled us to investigate how cells adapt to decreased subcellular NAD concentrations. For this purpose, we matched reaction fluxes to their corresponding pathways using MitoCore’s annotation [38]. We excluded a few pathways that were either artificial or biologically not meaningful, and merged others (refer to Table S1 for details). Subsequently, we calculated the mean of the absolute fluxes within each pathway. We focused on pathways with the highest standard deviation (σ) in mean absolute flux across cell lines. Then, we calculated the change in mean absolute pathway flux between the cell lines and the HEK293 high NAD model as

(4) $$\underline{c}=\left\|\frac{v^{\prime }-v}{v}\right\|*100$$

where v represents the flux in the HEK293 model with high NAD levels and v′ represents the flux in the other model. We capped changes at 1000% to accommodate cases where v=0. Finally, we extracted the active reactions from the most changed pathways (σ>0.01).

## 3. Results

**Figure 1:**
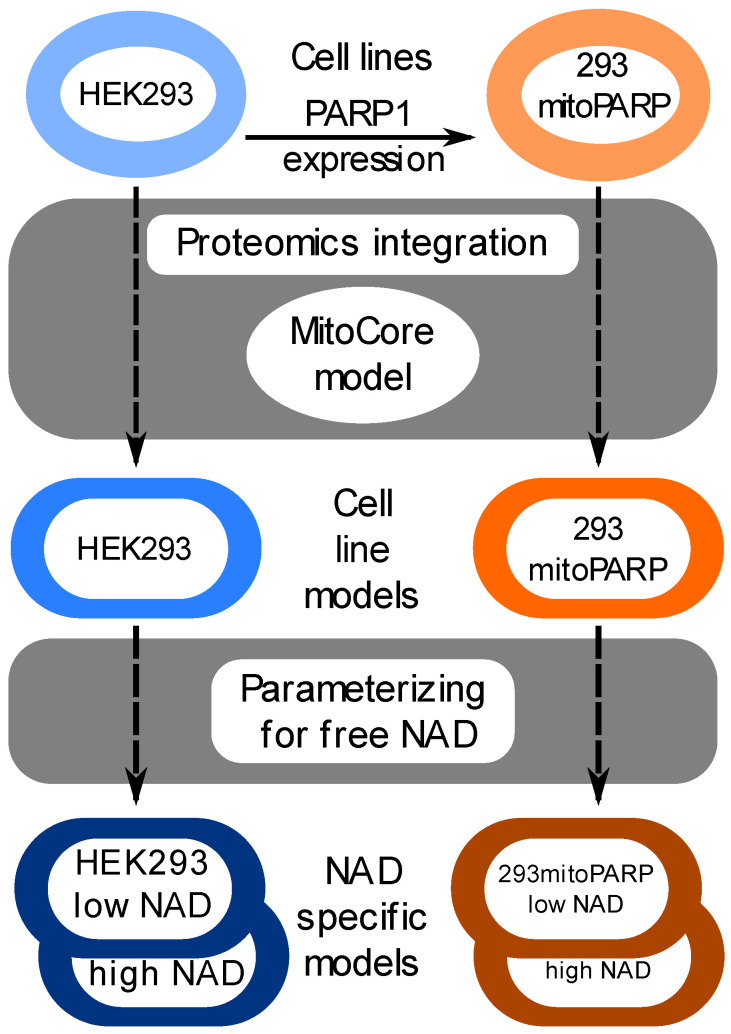
Workflow used to generate cell-line-specific models. The MitoCore [38] model was used to integrate proteome data from parental HEK293 and 293mitoPARP cell lines to generate cell-line-specific models using the GIMME [46] (see Section 2 for details). For each of these cell line models, a model parameterized for low NAD (NAD levels decreased to 60% in the cytosol and 10% in the mitochondria compared to parental) and for high NAD (parental) concentrations were generated.

To develop cell-line-specific metabolic models, we used the proteomics data from parental HEK293 and 293mitoPARP cells and integrated them into the MitoCore [38] model using the GIMME [46] algorithm (see Section 2 for details). The resultant cell-line-specific model of HEK293 and 293mitoPARP constitutes 470 and 462 reactions, respectively. 293mitoPARP is an engineered HEK293 cell line that constantly overexpresses the catalytic subunit of the NAD consumer Poly [ADP-ribose] polymerase 1 (PARP1) with a mitochondrial targeting signal [40]. This results in the chronic depletion of NAD [40]. The free NAD concentration has been measured to be approximately 230 μM in the mitochondria and 110 μM in the cytosol of parental HEK293 cells [52], whereas the 293mitoPARP cell line displays an up to 40–50% decrease in cellular NAD [41] and a severe depletion of NAD in mitochondria with only 10–20% of the mitochondrial NAD remaining [41] compared to parental HEK293 cells. The free NAD concentration in 293mitoPARP was thus decreased to approximately 66 μM in the cytosol and 23 μM in the mitochondria [41]. To model the changes related to free NAD concentrations, we retrieved $K_{m}$ values from the Brenda [49] enzyme database and adjusted the flux boundaries (cf. Equations (1)–(3)) in the model based on both measured NAD concentrations and the $K_{m}$ for the involved NAD species (see Section 2 for details). This resulted in the generation of NAD-concentration-specific models. An overview of our approach is shown in Figure 1. Next, we performed pFBA on the NAD-concentration-specific models to identify the minimal flux solution and the most important pathways for achieving the objective under the given free NAD concentrations.

To investigate the effects of NAD concentrations, we simulated four different conditions, including two real-life scenarios: (1) parental HEK293 protein abundance with parental free NAD concentrations (referred to as high in Figure 1); and (2) 293mitoPARP protein abundance with decreased free NAD (referred to as low in Figure 1) as measured in the corresponding cell line, and two hypothetical scenarios: (3) parental HEK293 protein abundance with low free NAD concentrations, which could represent a sudden unmitigated drop in NAD concentrations; and (4) 293mitoPARP protein abundance with parental NAD concentrations, which could represent cells adjusted to NAD depletion suddenly being exposed to high NAD concentrations—simulating the situation of NAD precursor supplementation in a system with chronic NAD depletion.

### 3.1. Accounting for NAD Concentrations Improves Predictions

**Figure 2:**
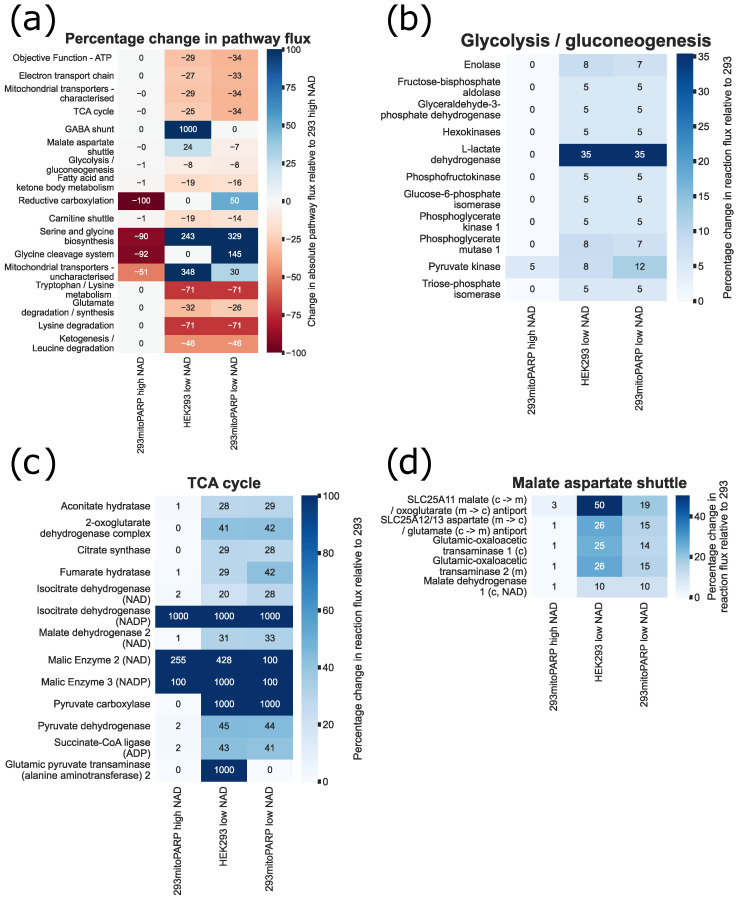
Simulation results for the most affected pathways. Only reactions with a standard deviation in fluxes of at least 0.01 are shown. (**a**) Percentage change in the mean absolute flux of each pathway relative to HEK293 with parental NAD levels. Positive numbers indicate an increase in pathway flux, while negative numbers indicate a decrease. Refer to Supplementary Figure S1 for absolute pathway fluxes. (**b**) Percentage change in reaction fluxes through glycolysis and gluconeogenesis. Changes in fluxes for reactions are shown as absolute changes. (**c**) Percentage change in reaction fluxes through the TCA cycle. (**d**) Percentage change in reaction fluxes through the malate–aspartate shuttle. Refer to Supplementary Figure S2 for the absolute changes in reaction fluxes.

Lowered NAD concentrations disrupted several key metabolic pathways, ultimately hindering the cells’ capacity to produce ATP. The pFBA solutions demonstrated a decrease of 29% and 34%, respectively, in maximum ATP production in both the HEK293 and 293mitoPARP models when the NAD concentration was lowered compared to the HEK293 model with the parental free NAD concentration (Figure 2a). Meanwhile, the ATP production was unchanged for the 293mitoPARP model with the parental NAD concentration (Figure 2a). When NAD concentrations are compromised, we predicted a decrease in flux through the electron transport chain, TCA cycle, and glycolysis (Figure 2a). Due to the reduced flux through the TCA cycle, an increase in flux through the GABA shunt was observed in the HEK293 model with the lowered NAD. In addition, we predicted a decreased flux through fatty acid oxidation and ketone body utilization pathways at lowered NAD concentrations (cf. Figure 2a). Our pFBA solutions showed that reduced NAD levels resulted in a decrease in carnitine shuttle activity. These findings align with the experimentally observed accumulation of medium-chain fatty acids [41]. Furthermore, the simulation results of the 293mitoPARP model predicted a decrease in the oxygen uptake (cf. Figure S2a), which is consistent with the experimental observations showing a decrease in the oxygen consumption rate in 293mitoPARP cells [41,42]. We also predicted an increase in lactate production owing to a decreased flux through the reaction catalyzed by lactate dehydrogenase at low NAD concentrations for both the parental HEK293 and 293mitoPARP models (Figure 2b). This was experimentally observed in 293mitoPARP cells [49,52] as medium acidification by lactate [40,41].

### 3.2. Metabolic Alterations in HEK293 Cells under Decreased NAD Concentrations

In addition to analyzing experimentally measured changes, we have also investigated alterations in other metabolic pathways encompassed within the model. We, for example, predicted that alanine aminotransferase (glutamic pyruvate transaminase 2) would carry zero flux under most conditions but that the absolute flux would increase in the HEK293 low NAD model. Alanine aminotransferase produces alanine and α-ketoglutarate from pyruvate and glutamate (Figure 2c). Furthermore, several pathways from amino acid metabolism were predicted to undergo alterations, including serine and glycine biosynthesis, and tryptophan and lysine metabolism, as well as leucine degradation. Many of these pathways were only affected when NAD levels were decreased in the models (Figure 2a). Malic enzyme 2 and 3, as well as pyruvate carboxylase, were predicted to carry increased flux in HEK293 at low NAD concentrations, while malate dehydrogenase showed lowered fluxes (Figure S2b). Activity in the malate–aspartate shuttle increased considerably in both HEK293 and 293mitoPARP with low NAD concentrations (Figure 2a,d), with the largest effect observed in the malate–ketoglutarate antiporter. It is interesting to note that pyruvate carboxylase fluxes at high NAD levels were predicted to be zero, while both HEK293 and mitoPARP, at low NAD levels, had significant fluxes with higher fluxes in HEK293. Similarly, the malic enzyme 2 flux was also more strongly affected in HEK293 than in 293mitoPARP at low NAD levels (Figure 2c). Taken together, several of the results point to a compensatory expression change in 293mitoPARP that led to a redirection of pathway fluxes to overcome the chronic depletion of NAD.

## 4. Discussion

It has been shown that a more realistic representation of single cofactors in CBMs can significantly enhance predictions [39]. Therefore, we analyzed pFBA solutions to investigate the impact of concentration changes in the most common metabolic cofactor, NAD, on reaction fluxes in the core metabolism. By defining an objective function, i.e., maximizing ATP production, pFBA predicted the optimal flux distribution through the metabolic network to achieve a given fraction of that objective. This enabled us to explore how the cell might adjust its metabolism under different free NAD concentrations.

NAD plays a pivotal role in numerous biological processes. Alterations in its concentration and turnover are a hallmark of many age-related diseases including metabolic diseases such as diabetes and obesity [54,55,56]. Understanding the cellular consequences of changes in NAD availability is vital for promoting healthy aging and advancing treatments for age-related conditions. Here, we adopted a systems biology approach by integrating free NAD concentrations into a core model of human metabolism. To accomplish this, we implemented flux boundary adjustments using Michaelis–Menten formulations and incorporated measured NAD concentrations. We used the MitoCore [38] model, a curated constraint-based model of human metabolism, to integrate proteomics data from both parental HEK293 and 293mitoPARP cells. Through the integration of proteomics data, we constructed cell-line-specific models of core metabolism. Additionally, we parametrized our cell-line-specific models to represent both parental and low levels of free NAD, enabling us to simulate metabolic behavior under varying NAD concentrations. Our predictions for the cell lines closely aligned with experimental observations. Notably, we accurately predicted the lowered respiratory rate and oxygen consumption, as well as medium acidification [40,41,42]. Furthermore, we showed that the changes in ATP demand and production in 293mitoPARP cells do not appear to be solely attributed to expression changes, as integrating proteomics data at parental NAD levels minimally affected ATP production.

We, here, presented a modeling approach that enables the integration of measured NAD concentration changes alongside expression data to predict genome-scale metabolic alterations. As a case study, we used a cellular system with chronic NAD depletion and showed that the cells adapted the expression of critical pathways to counterbalance some of the effects of chronic NAD depletion. We demonstrated that, in order to capture the experimentally observed metabolic changes, one explicitly needs to integrate lowered NAD concentrations by adjusting flux boundaries of NAD- and NADP-dependent reactions. We furthermore simulated the potential effect of NAD precursor supplementation in this cell system with chronic NAD depletion predicting genome-scale metabolic alterations that provide insights into potential systemic effects of this treatment. Moreover, we analyzed the hypothetical scenarios of sudden NAD depletion in wild-type cells, which would be challenging to perform experimentally. Our modeling approach accurately predicts the key behavior observed in NAD-depleted cell lines and highlights the importance of considering NAD concentrations in genome-scale modeling approaches to capture metabolic alterations under conditions of changed free NAD.

## 5. Conclusions

Modeling approaches enable the detailed analysis and exploration of biological systems under conditions and to an extent that is often not feasible in experimental settings. All modeling approaches are, however, simplified representations of the real world and, therefore, need to make assumptions and simplify the biological system they describe. One common assumption made in metabolic modeling approaches is the constant and non-changing availability of metabolic cofactors such as NAD(H) and NADP(H). But, as we know that changes in cofactor availability contribute to several diseases, we developed an approach to integrate measured changes in NAD(H) or NADP(H) concentrations into genome-scale modeling approaches. The resulting predictions are in good agreement with experimental results from a cell line stably overexpressing the catalytic domain of PARP targeted to mitochondria, representing a system with the long-term depletion of NAD. Our approach can be used to simulate disease-related or pharmacologically induced NAD depletion or to predict effects of NAD supplementation strategies. The predicted metabolic alterations could, in turn, be used to design targeted metabolomics measurements for disease characterization or treatment response monitoring.

## Figures and Tables

**Table 1:** NAD concentrations used to parameterize the different models. All values are in mM.

Cell Line	C_mitochondria_	C_cytosol_
HEK293	0.23	0.11
293mitoPARP	0.023	0.066

## Data Availability

The code created for the analysis, as well as the protein abundance ratios used, can be found at https://github.com/MolecularBioinformatics/NAD-GSMM, (accessed on 18 May 2024).

## Supplementary Materials

The following supporting information can be downloaded at: https://www.mdpi.com/article/10.3390/biom14050602/s1. Table S1: List of pathways dropped or merged for the pathway analyses. Figure S1: Mean absolute pathway flux in the cell lines. Figure S2: Absolute change in reaction fluxes through different metabolic pathways. Figure S3: Impact of the fraction of optimum used for creating cell-line-specific models in GIMME. Figure S4: Impact of decision function used for NAD integration. Figure S5: Impact of fraction of optimum used for NAD integration. Figure S6: Distribution of $K_{m}$ values extracted from Brenda and Sabio-RK enzyme databases.
