# Supplementary material for: Accounting for NAD Concentrations in Genome-Scale Metabolic Models Captures Important Metabolic Alterations in NAD-Depleted Systems
Source: Biomolecules. 2024 May 20;14(5):602. doi: 10.3390/biom14050602 (PMC11117748; doi:10.3390/biom14050602)
Supplement: Supplementary file 1 [file biomolecules-14-00602-s001.zip › biomolecules-2973713-supplementary.pdf]

Supplementary Material to **Accounting for NAD Concentrations in Genome-Scale Metabolic Models Captures Important Metabolic Alterations in NAD-Depleted Systems**

Table S1. List of pathways dropped or merged for the pathway analyses. For the analysis of changed pathways and reactions, we excluded some biologically not meaningful, or artificial pathways from the displayed results, and merged other pathways to avoid misrepresentations due to including mutually dependent or closely related pathways multiple times in our analyses. This did not change the function of the model itself, but only influences the results of the summary analysis.

**Pathway**	**Excluded or merged into**
Boundary Conditions - Biomarkers	excluded
Boundary conditions - core	excluded
Boundary Conditions - Misc	excluded
Cytosolic misc	excluded
Mitochondrial transport - diffusion / artificial	excluded
FA metabolism	FA and ketone body metabolism / Ketogenesis
Ketone bodies - degradation	FA and ketone body metabolism / Ketogenesis
Glycolysis	Glycolysis/gluconeogenesis
Glutamate degradation/synthesis - cytosolic	Glutamate degradation/synthesis
GABA shunt - transport	GABA shunt
Reductive carboxylation	Reductive carboxylation / Acetyl-CoA production for biosynthesis
TCA cycle periphery	TCA cycle

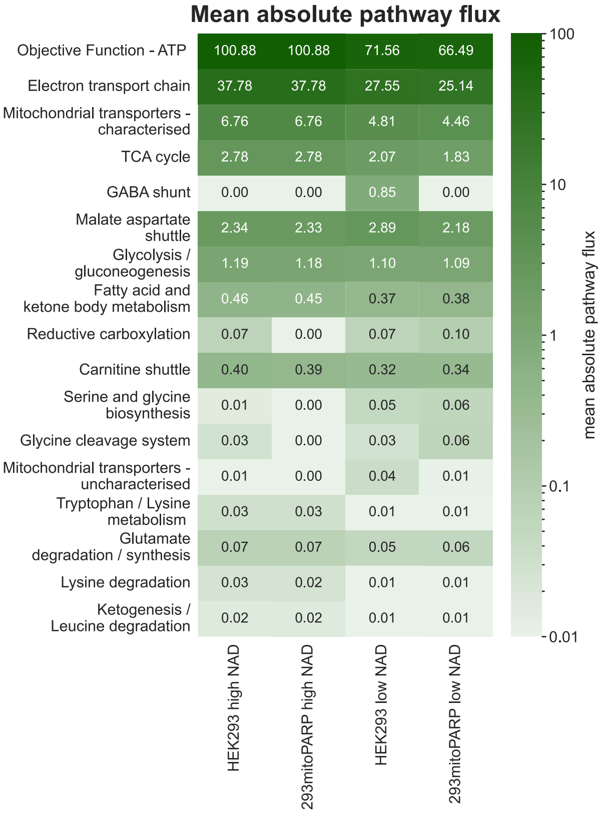

Figure S1. Mean absolute pathway flux in the cell lines. We performed pFBA on the cell line models, with ATP demand as the objective function. We then calculated the mean absolute flux of all reactions within the relevant pathways. The 20 pathways with the highest standard deviation across models in the resulting mean absolute flux between cell lines are shown. The method of calculation is described in Section 2. Pathways are excluded or merged as per Table S1.

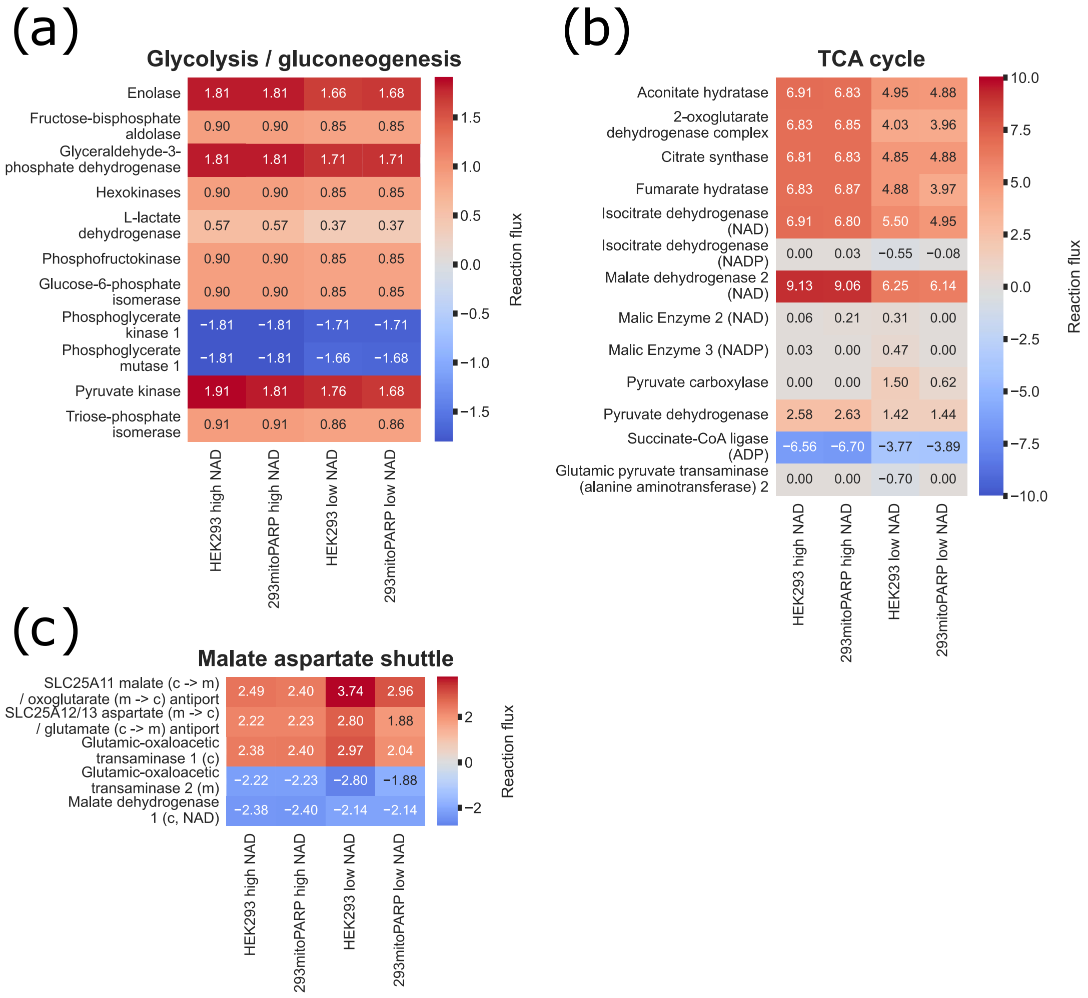

Figure S2. Absolute change in reaction fluxes through different metabolic pathways. We performed pFBA with maximum ATP demand as the objective on the 293mitoPARP and HEK293 models to extract reaction fluxes. Showing reactions out of selected pathways from the top 20 most changed pathways. All reactions within the pathways are shown if they display a standard deviation greater than 0.01 across all the cell lines viewed. Panel (a) Fluxes in the Glycolysis and gluconeogenesis pathway (b) Fluxes in the TCA cycle (c) Fluxes in the Malate aspartate shuttle.

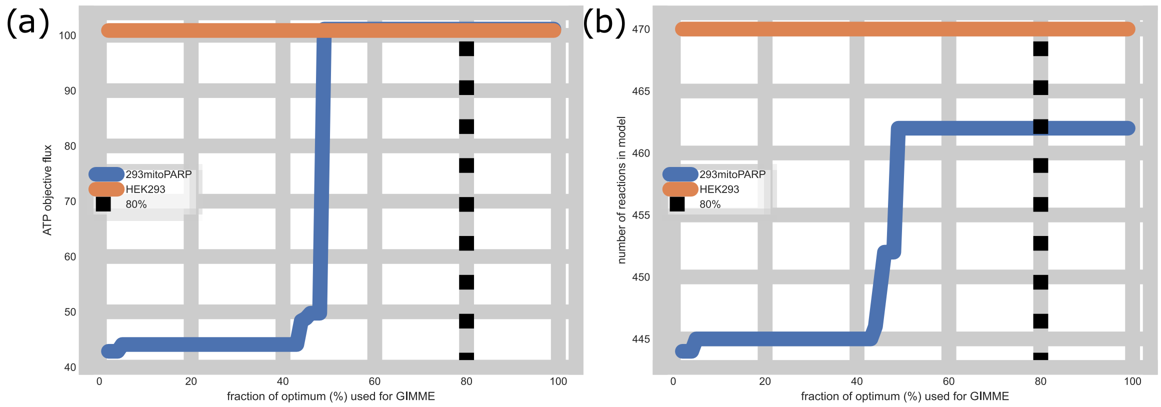

Figure S3. Impact of the fraction of optimum used for creating cell-line specific models in GIMME. The fraction used in the analyses is shown as a black dotted vertical line. (a) Impact on the ATP objective flux. The fraction of optimum used has no impact on the objective (ATP demand) in HEK293. In 293mitoPARP, it has impact only below ca. 50%, where it drops drastically, an effect not seen in the 293mitoPARP cell line. (b) Number of reactions in the models created at a given fraction of optimum. There is no impact on the number of reactions in the final model for HEK293. In 293mitoPARP there is some effect, but only below ca. 50%.

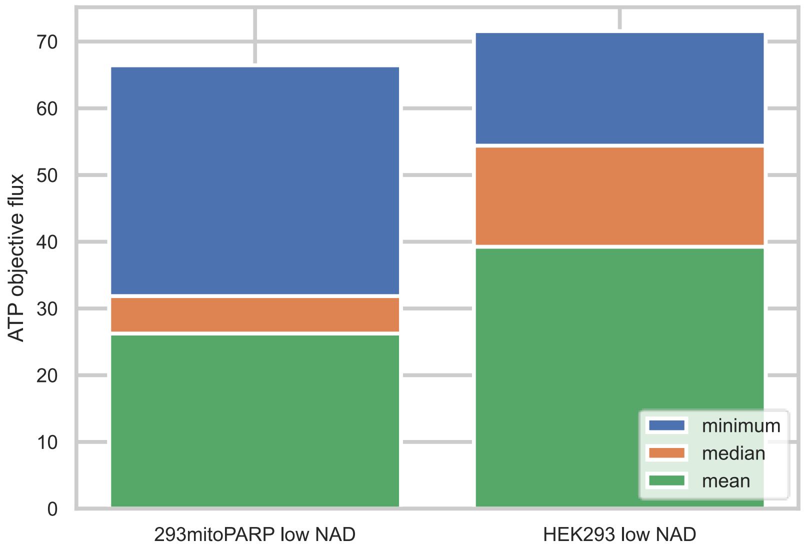

Figure S4. Impact of decision function used for NAD integration. Models for both 293mitoPARP and HEK293 were developed utilizing various decision functions to select among multiple mapped K_m values. In the analyses presented in the main text, the minimum function was employed. This means that if multiple Km values were mapped to the same reaction, the minimum K_m was chosen. It must be noted that the bars are not stacked, but the total height reached by each color represents the flux (mean = arithmetic mean).

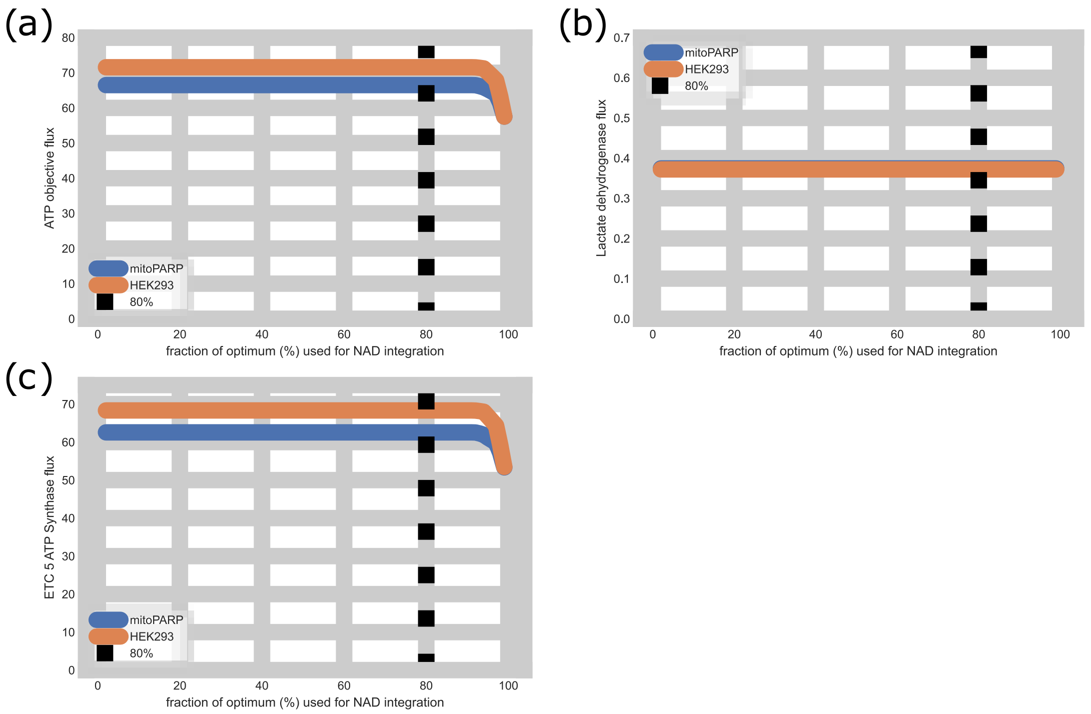

Figure S5. Impact of fraction of optimum used for NAD integration. Impact of the fraction of optimum used when parameterizing the models for a given NAD concentration in the HEK293 and 293mitoPARP models. Models are parameterized using the concentrations in Table 1 of the main text. Dotted vertical lines indicate 80% of optimum. Objective value (ATP demand) in the models. Fractions of optimum ≥ 80% do not affect ATP production in the 293mitoPARP model. In the HEK293 model this effect is even more pronounced with optima up to and around 90% having no effect.

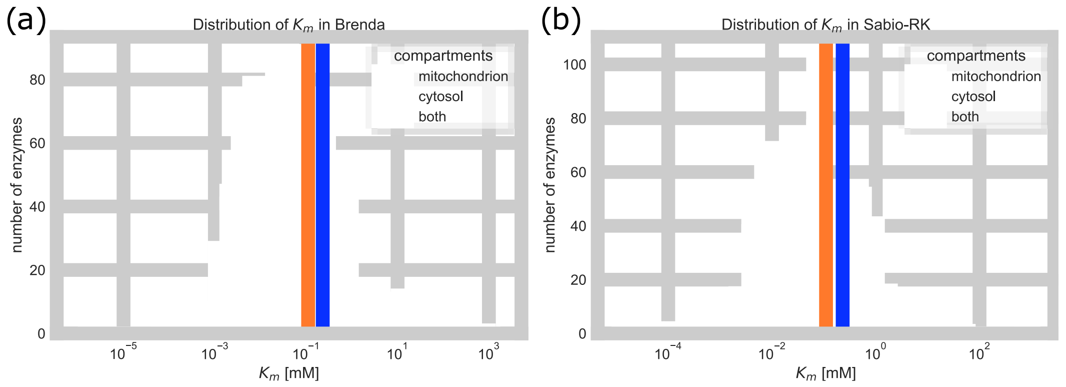

Figure S1. Distribution of K_m values extracted from Brenda and Sabio-RK enzyme databases The distribution of K_m is shown in the histograms, grouped by the location of the reaction the enzymes mapped to. Healthy wild-type NAD concentrations in mitochondria (blue) and cytosol (orange) are shown as vertical lines. In both SabioRK and Brenda, most K_m found are around the healthy wild-type concentrations, indicating that most enzymes are not saturated in wild-type conditions. The same applies to the subset of K_m mapping to either compartment. (a) Distribution of K_m extracted from Brenda (b) Distribution of K_m extracted from SabioRK.
